# Prediction of Novel Anoctamin1 (ANO1) Inhibitors Using 3D-QSAR Pharmacophore Modeling and Molecular Docking

**DOI:** 10.3390/ijms19103204

**Published:** 2018-10-17

**Authors:** Yoon Hyeok Lee, Gwan-Su Yi

**Affiliations:** Department of Bio and Brain engineering, Korea Advanced Institute of Science and Technology (KAIST), 291 Daehak-ro, Yuseong-gu, Daejeon 34141, Korea; yoonhuk30@kaist.ac.kr

**Keywords:** anoctamin1 (ANO1), pharmacophore, three-dimensional quantitative structure-activity relationship (3D-QSAR), molecular docking, virtual screening

## Abstract

Recently, anoctamin1 (ANO1), a calcium-activated chloride channel, has been considered an important drug target, due to its involvement in various physiological functions, as well as its possibility for treatment of cancer, pain, diarrhea, hypertension, and asthma. Although several ANO1 inhibitors have been discovered by high-throughput screening, a discovery of new ANO1 inhibitors is still in the early phase, in terms of their potency and specificity. Moreover, there is no computational model to be able to identify a novel lead candidate of ANO1 inhibitor. Therefore, three-dimensional quantitative structure-activity relationship (3D-QSAR) pharmacophore modeling approach was employed for identifying the essential chemical features to be required in the inhibition of ANO1. The pharmacophore hypothesis 2 (Hypo2) was selected as the best model based on the highest correlation coefficient of prediction on the test set (0.909). Hypo2 comprised a hydrogen bond acceptor, a hydrogen bond donor, a hydrophobic, and a ring aromatic feature with good statistics of the total cost (73.604), the correlation coefficient of the training set (0.969), and the root-mean-square deviation (RMSD) value (0.946). Hypo2 was well assessed by the test set, Fischer randomization, and leave-one-out methods. Virtual screening of the ZINC database with Hypo2 retrieved the 580 drug-like candidates with good potency and ADMET properties. Finally, two compounds were selected as novel lead candidates of ANO1 inhibitor, based on the molecular docking score and the interaction analysis. In this study, the best pharmacophore model, Hypo2, with notable predictive ability was successfully generated, and two potential leads of ANO1 inhibitors were identified. We believe that these compounds and the 3D-QSAR pharmacophore model could contribute to discovering novel and potent ANO1 inhibitors in the future.

## 1. Introduction

Anoctamin1 (ANO1/TMEM16A) is a calcium-activated chloride channel (CaCC) that responds to an increase of intracellular Ca^2+^ concentration [1,2,3]. Since the time when the molecular identity of ANO1 was deciphered by the three independent groups in 2008 [1,2,3], various aspects of physiological and pathological relevance of ANO1 have been discovered up to now. ANO1 is ubiquitously expressed in many tissues [4] and it is known to play important roles in fluid secretion, smooth muscle contraction, nociception, insulin secretion, cell proliferation, and migration [5,6]. In addition, ANO1 has emerged as a new drug target for the treatment of cancer, pain, diarrhea, hypertension, and asthma [5,6,7].

Despite the important biological role of ANO1, a discovery of new ANO1 inhibitors is still in the early phase. Due to the absence of structural information of ANO1 until 2017, most of the ANO1 inhibitors have been discovered through high-throughput chemical library screening using a yellow fluorescent protein (YFP)-iodide based sensor [7,8]. To date, several ANO1 inhibitors such as CaCC_inh_-A01 [9], T16_inh_-A01 [10], MONNA [11], benzbromarone [12], Ani9 [13], tannic acid [14], eugenol [15], luteolin [16], and crofelemer [17] have been discovered from both the chemical and natural product space. Of these, CaCC_inh_-A01, T16_inh_-A01, MONNA, and Ani9 are the most potent chemical inhibitors of ANO1, whose half maximal inhibitory concentration (IC_50_) values range from 100 nM to 3 μM [9,10,11,13]. Natural product inhibitors have IC_50_ values up to 10 μM or more, which are higher than those of chemical inhibitors [14,15,16,17]. Among the natural product inhibitors of ANO1, the crofelemer (previously known as Fulyzaq and now as Mytesi) from Napo Pharmaceutical in 2012 is the first FDA-approved drug to be used for anti-human immunodeficiency virus (HIV) associated anti-diarrhea, through targeting the ANO1 [17,18].

Although many ANO1 inhibitors have been experimentally discovered, most of the ANO1 inhibitors still have revealed a low potency and selectivity (μM level) [5]. In addition, many ANO1 inhibitors have revealed the inhibition of the structurally similar ANO2 (62% of amino acid homology) [3,19], and also the other ion channels such as CFTR, ENaC, and BEST1 [5,20]. Therefore, there is a need to find more potent and selective inhibitors as novel lead candidates targeting ANO1. Although high-throughput chemical library screening has been successfully explored several ANO1 inhibitors so far, it is very labor intensive and has a low hit-rate compared to the effort required. In addition, there is still no available structural information regarding the discovery of novel leads of ANO1.

We aimed to generate a chemical feature-based pharmacophore model for identifying novel lead candidates with the potential to be ANO1 inhibitors. The pharmacophore model contains abstract features that define interaction types that are necessary for chemicals’ biological activities [21]. Thus, the virtual screening of a chemical library using the pharmacophore model could usually guide the design of novel lead candidates. A ligand-based pharmacophore modeling approach with subsequent molecular docking study has identified several novel lead candidates of renin, tubulin, PDE4, BACE1, AKR1B10, and so on [22,23,24,25,26]. Moreover, the structures of mouse ANO1 have been elucidated recently by cryo-electron microscopy (cryo-EM) techniques [27,28,29]. Thus, both ligand-based and structure-based drug discovery approaches are able to be applied for identifying the novel ANO1 inhibitor candidates.

In this study, a three-dimensional quantitative structure–activity relationship (3D-QSAR) pharmacophore model was generated based on the chemical features present in known ANO1 inhibitors. Then, virtual screening of large chemical database (ZINC) was carried out to obtain novel lead candidates using the pharmacophore model. The retrieved hit compounds were subjected to molecular docking for further characterization. Finally, final hit compounds with a favorable binding mode to ANO1 were identified as novel lead candidates.

## 2. Results and Discussion

### 2.1. Pharmacophore Model Generation

**Figure 1:**
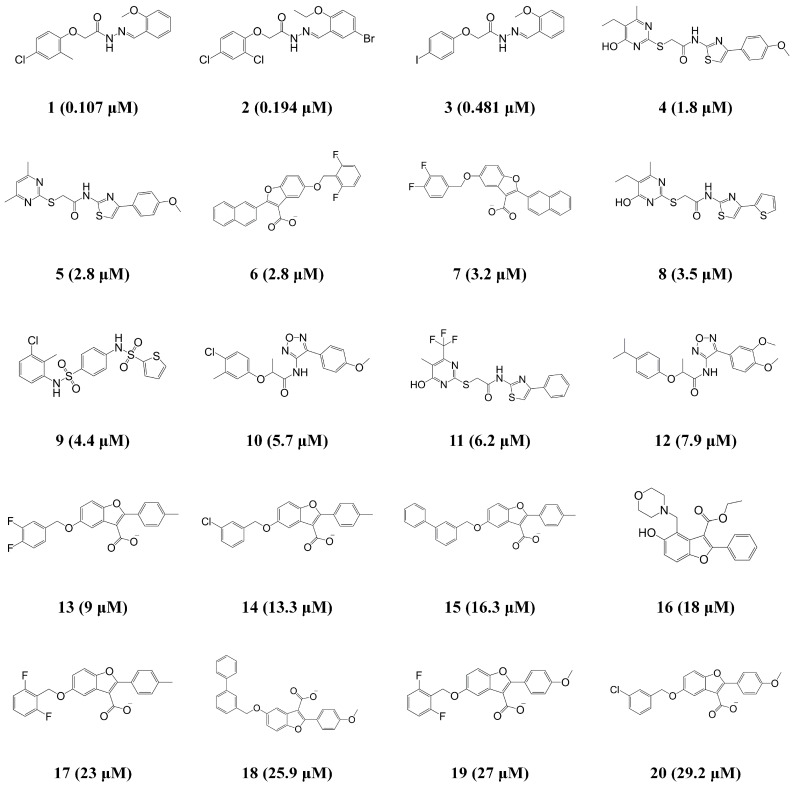
Two-dimensional (2D) chemical structures of the 20 training set compounds with their experimental half maximal inhibitory concentration (IC_50_) values.

The HypoGen algorithm in Discovery Studio 3.1 (DS) from Accelrys (San Diego, CA, USA) was implemented to generate the 3D-QSAR pharmacophore model that can quantitatively predict the biological activity (IC_50_) of given compounds against ANO1. The 20 compounds with activities ranging from 0.107 to 29.2 μM in the training set were used to train the pharmacophore models (Figure 1). For identifying the meaningful pharmacophore features from the training set, the Feature mapping of DS was carried out, and it resulted in hydrogen bond donor (HBD), hydrogen bond acceptor (HBA), hydrophobic (HY), and ring aromatic (RA) features. As a result of the HypoGen algorithm with the training set and their pharmacophore features, 10 top-scored hypothetical pharmacophore models were generated with their statistical parameters (Table 1).

Then, a cost analysis was conducted to assess the quality of the generated pharmacophore hypotheses. We used the following values for assessing the hypotheses: (i) the cost difference between null cost and fixed cost; (ii) the cost difference between null cost and total cost; (iii) the configuration cost; (iv) the root-mean-square deviation (RMSD); (v) the correlation coefficient of the training set (r^2^_training_). First, the 10 hypotheses were developed with the fixed cost of 63.804 and the null cost of 194.608. The larger cost difference (130.804) between the null cost and the fixed cost implied that the first and second models (Hypo1 and Hypo2) are meaningful models that have more statistical significance than 90%. Second, the total cost of the 10 pharmacophore hypotheses ranged from 70.969 to 87.091. Among them, the total cost of Hypo1 and Hypo2 were calculated to be closer to the fixed cost than other models. The cost differences between the null cost and the total cost of the Hypo1 and Hypo2 were 123.64 and 121.00, respectively. Third, the configuration cost in this study was 15.343, which would be less than 17 [23]. This indicates that the correlation from the generated pharmacophores cannot be attributed to chance. Fourth, the RMSD values of the top two pharmacophore hypotheses were less than 1, which illustrates the good regression ability of these hypotheses. Finally, the r^2^_training_ values of the 10 pharmacophore models were greater than 0.9, and the top two models have the higher r^2^_training_ values above 0.95 indicating the strong capability to predict the biological activity of the training set compounds. Among the 10 hypotheses, eight of 10 hypotheses including top two hypotheses consist of HBA, HBD, HY, and RA features.

### 2.2. Pharmacophore Model Evaluation and Selection

**Figure 2:**
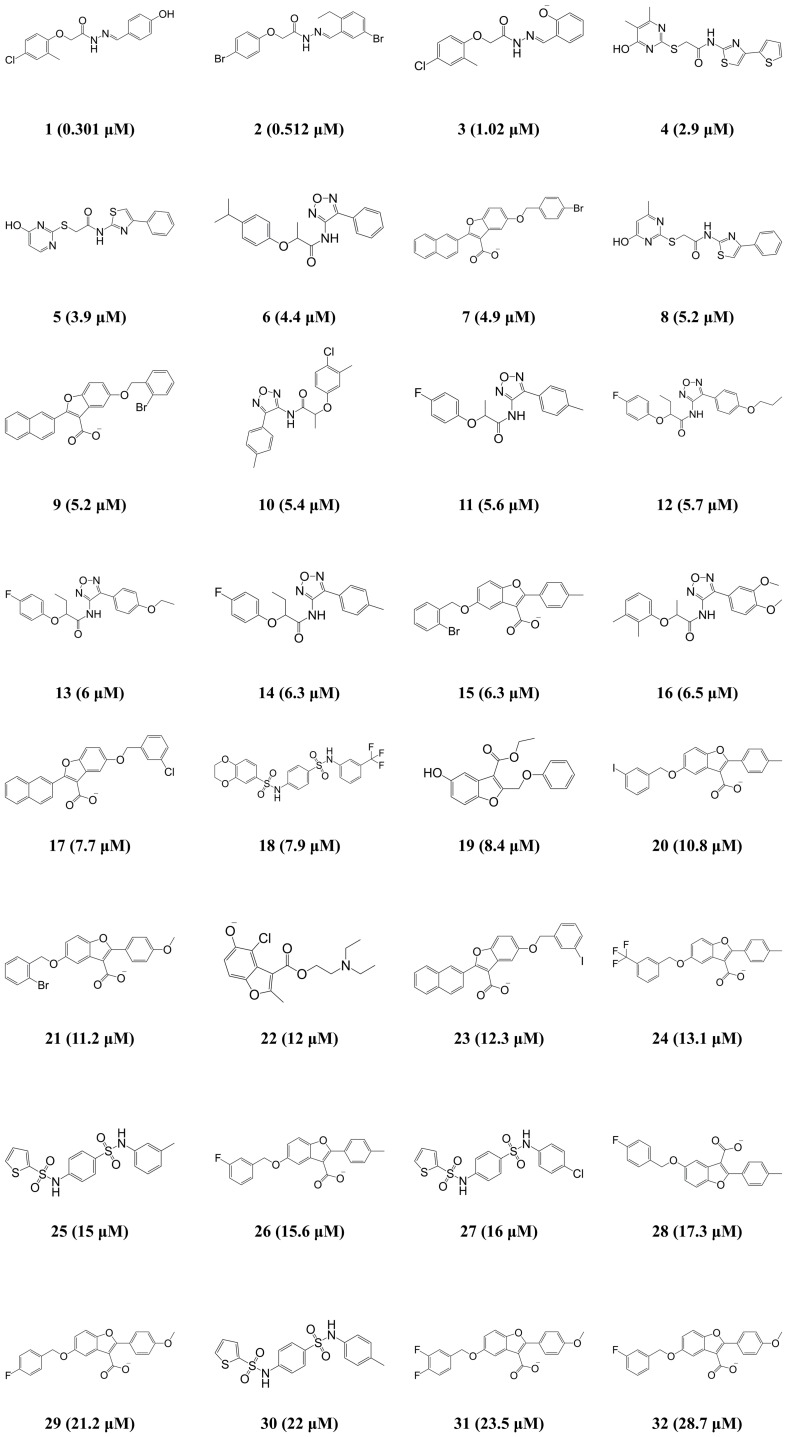
2D chemical structures of the 32 test set compounds with their experimental IC_50_ values.

**Figure 3:**
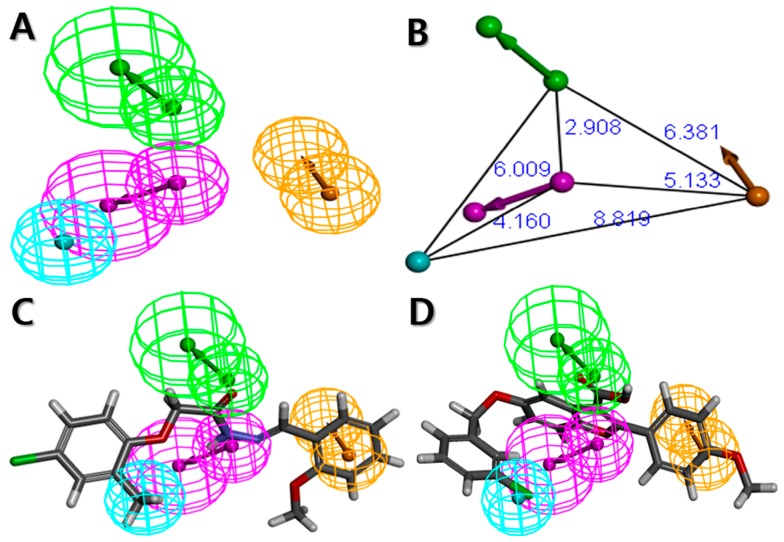
The best HypoGen pharmacophore model, Hypo2. (**A**) Pharmacophore features present in Hypo2; (**B**) 3D spatial relationship and geometric parameters of Hypo2; (**C**) Mapping of the most active compound (compound 1) on Hypo2; (**D**) Mapping of the least active compound (compound 20) on Hypo2. Pharmacophore features are color-coded: magenta, hydrogen bond donor (HBD); green, hydrogen bond acceptor (HBA); cyan, hydrophobic (HY); orange, ring aromatic (RA).

Based on the results of the cost analysis, the Hypo1 model should be selected as the best model for further analysis. However, the important factor in order to be the best pharmacophore model is that whether the generated pharmacophore model has the best ability to estimate the activity of new compounds which are retrieved from a database consisting of activity-unknown compounds. Therefore, the correlation coefficient of the test set (r^2^_test_) value was used as the primary criterion to select the most reliable pharmacophore model among the 10 generated hypotheses. The 32 compounds with activity ranging from 0.301 to 28.7 μM were selected as the test set (Figure 2). Diverse conformations of the test set compounds were prepared in the same manner as the training set preparation. To assess the regression ability of the generated pharmacophore models about the test set, each generated pharmacophore hypothesis was applied to predict the biological activity of the 32 test set compounds. As a result, the r^2^_test_ values of each hypothesis were calculated (Table 1), and only one hypothesis (Hypo2) revealed the high correlation coefficient of 0.909. Despite the best results of Hypo1 in the cost analysis, the Hypo1 model showed an r^2^_test_ value of 0.75. Compared to the cost analysis result of Hypo1, Hypo2 has similar statistical values in terms of the total cost (73.604), the cost difference (121.00), the RMSD (0.946), and the r^2^_training_ (0.969). Hence, Hypo2, which consists of each of HBA, HBD, HY, and RA was determined as the best hypothesis model for further analysis (Figure 3A). The inter-spatial constraints are shown in Figure 3B.

To further explore the model performance of Hypo2 on the training set, the training set compounds were primarily categorized into following four groups based on experimental activities: most active (IC_50_ < 1 μM, ++++), active (1 ≤ IC_50_ < 8 μM, +++), moderately active (8 ≤ IC_50_ < 18 μM, ++), and inactive (IC_50_ ≥ 18 μM, +). The activities and its scales of the training set compounds were then estimated based on Hypo2. As shown in Table 2, only one compound (compound 17) was estimated wrongly as being moderately active (++), which is overestimated than the activity scale of experimental value. The rest of the compounds were estimated perfectly as their corresponding activity scales of experimental values, indicating the good predictability of the Hypo2. In addition, none of the training set compounds had an error value above 2.5, signifying that the activity scale difference is below one order of magnitude. Error value indicates the ratio between the estimated and experimental activities. Then, pharmacophore features of the Hypo2 were mapped to the training set compounds. The mapping results of the most and the least active compounds on the Hypo2 are shown in Figure 3C and D, respectively. The most active compound (compound 1) were well fitted to all the pharmacophore features of the Hypo2, consisting of each one of HBA, HBD, HY, and RA, and the fit value was 5.9. On the other hand, the least active compound (compound 20) was mapped to the three features of the Hypo2, except for HBD, and the fit value was 3.58. These results signified that Hypo2 is a significant model to accurately predict the experimental activity of the training set compounds.

### 2.3. Pharmacophore Model Validation

To evaluate the best pharmacophore model (Hypo2) further, we used the test set, Fischer randomization, and leave-one-out methods.

#### 2.3.1. Test Set Method

**Figure 4:**
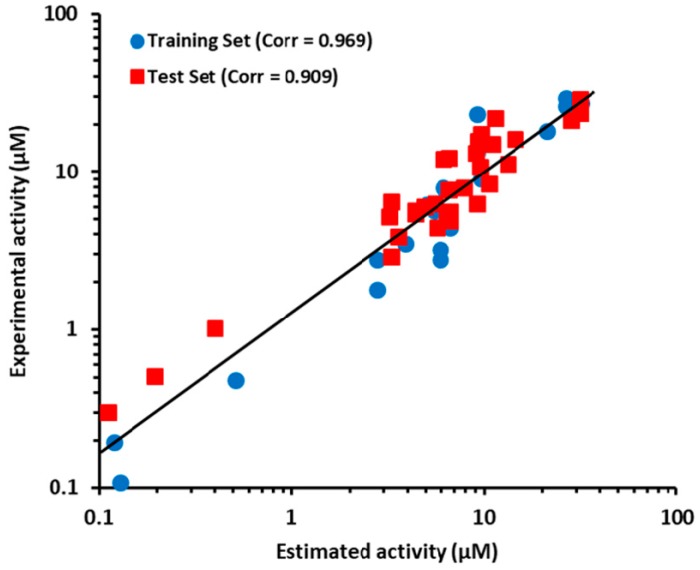
The correlation graph between experimental and estimated activity values for the training set and test set compounds based on Hypo2.

The prediction ability of Hypo2 had already evaluated at the best model selection step. The r^2^_test_ value of the regression using Hypo2 revealed value of 0.909 (Figure 4). The test set compounds were also categorized into the four groups, which were at the same activity range as the training set compounds. The activities and its scales of the test set compounds were then estimated based on Hypo2 as well. As shown in Table 3, 29 of the 32 test set compounds revealed error values of less than 2. Five compounds (compound 3, 15, 22, 23, and 30) out of 32 test set compounds were estimated in a different activity scale resulting in a prediction rate of 84.4%. Among these five compounds, compound 15 was only underestimated, while the rest of the four compounds were overestimated compared to the scale of experimental activity. This overestimation tendency of the Hypo2 model implied that the Hypo2 model might act to minimize the false-negative compounds, in spite of increment of false-positive candidates. According to the above results, these results suggested that Hypo2 has a good prediction performance of ANO1 inhibitors from newly queried compounds.

#### 2.3.2. Fischer Randomization Method

**Figure 5:**
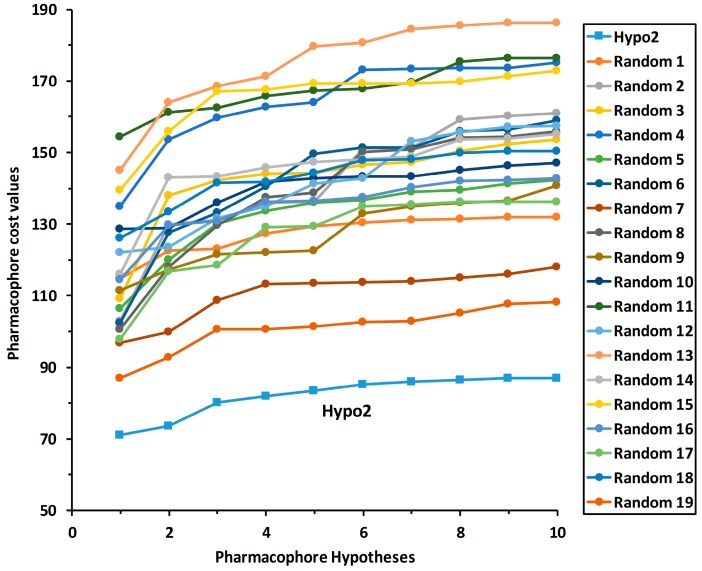
Fischer randomization test results of Hypo2 for 95% confidence level.

To validate the statistical significance of the Hypo2 model, the Fischer randomization method was carried out based on the randomized activity data of the training set compounds. This method could test that the pharmacophore model was not generated by the random correlation between biological activities and structures of training set compounds. To reveal that the Hypo2 model was not generated by chance with a 95% confidence level, 19 hypotheses were generated from spreadsheets consisting of random relationships between chemical structures and biological activities (Figure 5). None of the 19 randomly generated hypotheses scored a lower total cost than that of Hypo2. This Fischer randomization result clearly indicates that Hypo2 is statistically robust and not generated by chance because Hypo2 has represented a true correlation in the training set.

#### 2.3.3. Leave-One-Out Method

The leave-one-out (LOO) method was performed for validating Hypo2. The purpose of this validation method is to verify that the r^2^_training_ value of Hypo2 is not only dependent on one particular compound in the training set. To this end, the 20 pharmacophore hypotheses were regenerated under the same parameter setting of the Hypo2 by omitting one compound at a time from the training set. If there is no meaningful difference of r^2^_training_ value between Hypo2 and the newly generated hypothesis in the LOO step, the LOO test is passed. To assess the statistical significance of the Hypo2, a RMSD value between the r^2^_training_ of the Hypo2 (0.969) and the mean value of r^2^_training_ of each hypothesis from LOO was calculated. The RMSD of r^2^_training_ from the LOO step was 0.0112, which was very small value, signifying that there is no significant difference between Hypo2 and each hypothesis from LOO. This result suggested that the Hypo2 is a robust model that is not affected by only one particular compound in the training set. Based on the aforementioned results, Hypo2 was determined for use in identifying potent candidates to be ANO1 inhibitors from a large chemical database.

### 2.4. Virtual Screening

**Figure 6:**
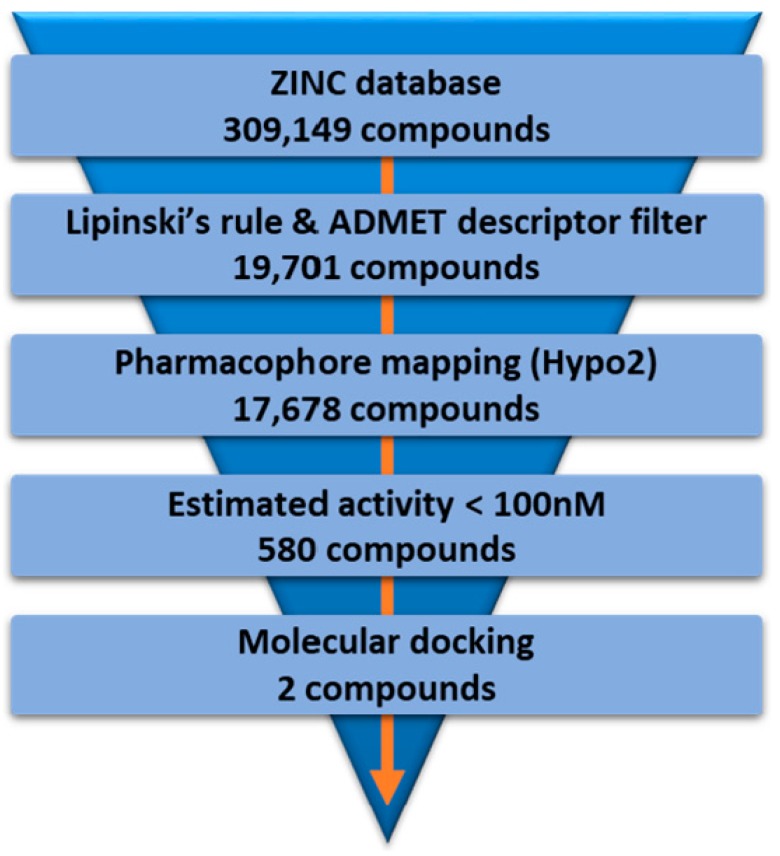
The flowchart of virtual screening using Hypo2.

The Hypo2 model was used to retrieve novel hit compounds from the ZINC database [32]. First, the Lipinski’s rule and the absorption, distribution, metabolism, excretion, and toxicity (ADMET) properties of 309,149 compounds were calculated. As a result of Lipinski’s rule and ADMET descriptor filtering, a total 19,701 compounds remained as drug-like candidates with good ADMET properties. Then, these filtered compounds were subsequently applied to the Ligand Pharmacophore Mapping of DS with the Hypo2 pharmacophore model. A total of 17,678 compounds were retrieved by pharmacophore mapping of the Hypo2. Among these 17,678 compounds, we finally selected 580 compounds as hit candidates by restricting the estimated activity to less than 100 nM. These 580 compounds that met all the above criteria were applied to molecular docking studies for further characterization. The flowchart of virtual screening is described in Figure 6.

### 2.5. Molecular Docking

To reduce the false-positive candidates from the results of ligand-based virtual screening and to refine the novel lead candidates, the retrieved hit compounds, along with the most active (++++) and active (+++) groups compounds in the dataset were docked into ANO1, using the Libdock algorithm in DS. Although the protein structure of ANO1 is recently deciphered [27,28,29], the binding mode of ANO1 inhibitors on ANO1 is still unclear. Since the ANO1 is a CaCC class channel, many researchers have focused on the putative residues of calcium-binding site through the mutagenesis studies for deciphering association between each residue and calcium-dependent activity changes of ANO1 [29,33,34,35,36]. The influences of the following mutants to the activity of ANO1 have been reported so far: K588, K645, E654, E702, E705, E734, and D738 residues [29,33,34,35,36]. Then, these residues finally have been identified as the core residues of calcium-binding site and intracellular vestibule site [27,28]. Therefore, in this study, the active site was defined, based on the vicinity of the calcium-binding site in the structure of ANO1.

**Figure 7:**
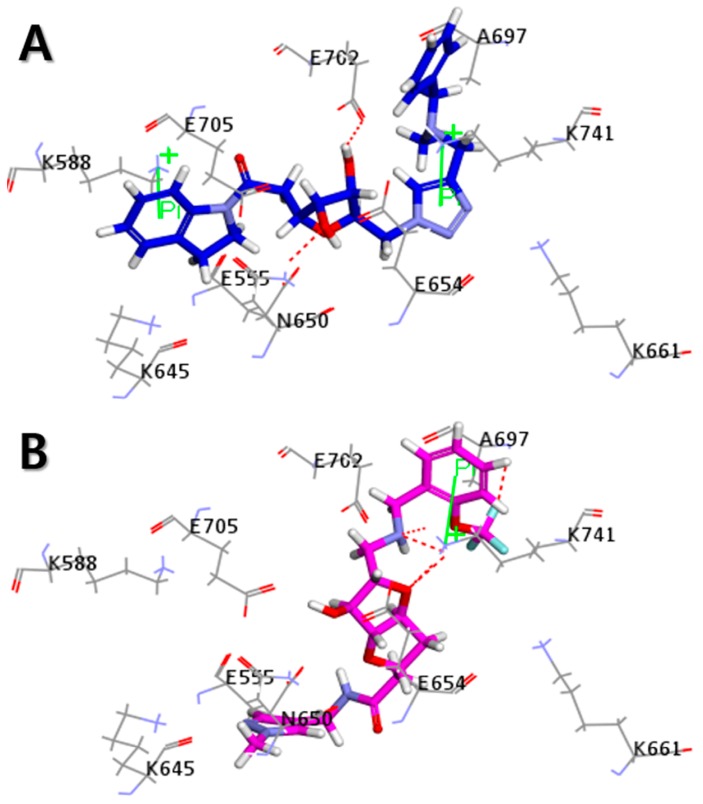
Molecular docking results. 3D interaction diagram for the calcium-binding site of ANO1 with (**A**) ZINC8643627 (blue) and (**B**) ZINC225516955 (magenta). Hydrogen bond interactions and Pi–cation interactions are described in red dotted line and green line, respectively.

**Figure 8:**
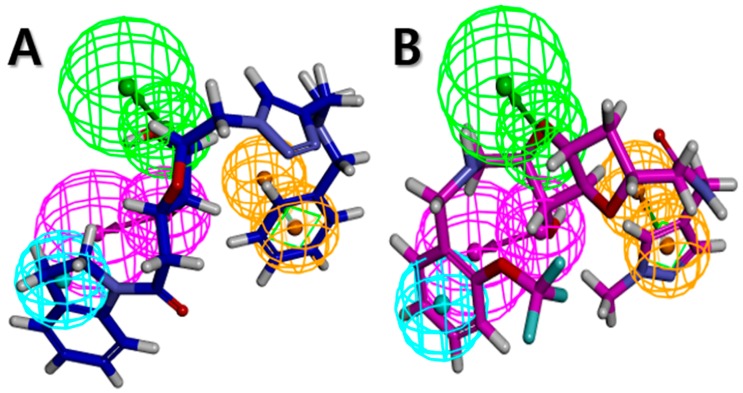
Pharmacophore mapping of two final hit compounds on Hypo2. (**A**) ZINC8643627 (blue) (**B**) ZINC225516955 (magenta). Pharmacophore features are color-coded: magenta, hydrogen bond donor (HBD); green, hydrogen bond acceptor (HBA); cyan, hydrophobic (HY); orange, ring aromatic (RA).

The Libdock scores (LS) were calculated for all 610 compounds, which consist of the 580 retrieved hit compounds along with the 30 compounds in the most active (++++) and active (+++) groups of the dataset. Interestingly, the most active (++++) group compounds including compound 1 of the training set, which were derived from the same scaffold structure, showed a much lower average LS value (102.35) compared to the average LS value of the other dataset compounds (114.49). This implied that the most active (++++) group compounds may interact with different binding sites for the strong inhibition of ANO1. The highest LS value of the dataset compounds was 135.61. To find a promising novel lead candidate of ANO1 inhibitors, hit compounds were selected based on the strict threshold greater than LS value of 155. As a result, only five compounds (ZINC8643627, ZINC225516955, ZINC8624466, ZINC225516884, and ZINC225519862) showed the LS values above 155. Among the five compounds, ZINC8643627 and ZINC8624466 had the same scaffold structure, and ZINC225516955, ZINC225516884, and ZINC225519862 had the same scaffold structure as well. Thus, we decided to analyze further only one compound per each scaffold structure, which had the highest LS value among the compounds of the same scaffold. Finally, two compounds (ZINC8643627 and ZINC225516955) with different scaffold structures were selected as lead candidate compounds of ANO1. ZINC8643627 and ZINC225516955 revealed LS values of 162.29 and 160.92, respectively. The binding modes of the two compounds are depicted in Figure 7. The calcium-binding site of ANO1 contains Q646, K650, N651, E654, E702, E705, N730, E734, and D738 [28]. The intracellular vestibule site of ANO1 contains E555, K588, K645, E654, E702, and K741 [28]. ZINC8643627 has formed hydrogen bond interactions with N650 and E702, which are the critical residues for calcium-ion binding according to the mutagenesis studies [33,34]. It has also formed Pi–cation interactions with K588 and K741, which are located in the intracellular vestibule site of ANO1 (Figure 7A). The ZINC225516955 formed hydrogen bond interactions with A697 and K741 as well as Pi–cation interaction with K741 (Figure 7B). The mapping results of the two final hit compounds on Hypo2 are shown in Figure 8. ZINC8643627 revealed a fit value of 6.29 and an estimated activity value of 0.052 μM. ZINC225516955 revealed a fit value of 6.04 and an estimated activity value of 0.092 μM. The 2D representations of the two final hit compounds with corresponding estimated scores are shown in Table 4. Interestingly, these two final hit compounds were both natural products, and they did not have any reports on the inhibitory activity of ANO1 based on the PubChem [37] search, signifying the novelty of the two final hit compounds. All of these results suggested that ZINC8643627 and ZINC225516955 may highly affect the calcium-binding and the intracellular vestibule site with low IC_50_ values, resulting in the current disturbance of ANO1.

## 3. Materials and Methods

### 3.1. Pharmacophore Model Generation

#### 3.1.1. Selection of Dataset Compounds

To represent the structural diversity and broad activity range, the 52 compounds were retrieved from the four literature resources [10,13,30,31]. To achieve a significant pharmacophore modeling, among the 52 dataset compounds with the experimental activity values (IC_50_), 20 compounds were selected as a training set and the remaining 32 compounds were used as a test set during pharmacophore model validation. The above dataset was selected based on the following criteria [25,38]: (1) All 52 compounds have an inhibitory activity against the ANO1 channel. The apical membrane current of all 52 compounds was determined by an ANO1 functional assay using Fischer rat thyroid (FRT) cells, which can stably express human ANO1 and the halide sensor YFP–H148Q/I152L/F46L. (2) The dataset compounds should be widely populated and less redundant. The IC_50_ values of both training set and test set compounds consist of a wide activity range from 0.107 to 29.2 μM. (3) To avoid using the different standard of IC_50_ values measured by different assays and research groups, all IC_50_ values of 52 compounds were collected from the same biological assays (YFP fluorescence plate reader assay) and the research groups (the A.S. Verkman group and their colleagues, UCSF, San Francisco, CA, USA).

#### 3.1.2. Dataset Compound Preparation

The 2D structures of all the compounds in the dataset were drawn by using ChemDraw Ultra 12.0 (Cambridge Soft Corp., Cambridge, MA, USA), and subsequently converted to 3D structures in DS. Then, hydrogen atoms were added to these 3D structures of compounds as their proper ionized forms under the pH environment of 7.4. Energy minimization of all compounds was carried out using the smart minimizer that carried out 1000 steps of the steepest descent algorithm, followed by a conjugate gradient algorithm with a convergence gradient of 0.001 kcal/mol. After the energy minimization step, the Poling algorithm in the Generate Conformations of DS with the Best conformer generation option under the Chemistry at Harvard Macromolecular Mechanics (CHARMM) force filed was used to generate multiple conformers. A maximum number of 255 conformations with an energy threshold of 10 kcal/mol above the global energy minimum were generated for each compound in the dataset [22,25,38].

#### 3.1.3. Generation of Pharmacophore Models

The HypoGen algorithm [38] in DS was used to perform the 3D-QSAR pharmacophore modeling. This algorithm can generate hypothetical pharmacophore models that correlate between a 3D spatial arrangement of features in a submitted training set compounds and their respective biological activities based on following three steps: the constructive phase, the subtractive phase, and the optimization phase [38]. For establishing the hypothetical pharmacophore model, the Feature mapping of DS was first used to identify the inherent pharmacophore features of the 20 training set compounds. The resulting features were used to generate 10 hypothetical pharmacophore models using the 3D-QSAR pharmacophore generation of DS. All other control parameters of the HypoGen algorithm were kept to their default settings, except for the uncertainty value and the minimum inter-feature distance, which were set to 1.5 and 2 Å, respectively [22,23].

### 3.2. Pharmacophore Model Evaluation

#### 3.2.1. Cost Analysis

The generated pharmacophore models were briefly assessed for their quality based on the following three cost parameters, which were calculated in the unit of bits during the pharmacophore model generation: the null cost, the fixed cost, and the total cost [38]. The null cost is equal to the maximum occurring error cost, which represents the highest cost value of a pharmacophore model with no features. The fixed cost is a cost of the simplest ideal model that can predict all the data points perfectly. The total cost represents the cost value of each generated pharmacophore hypotheses calculating by the summation of the weight cost, the configuration cost, and error cost. The details of the above three cost parameters are described in [22,38]. For a significant pharmacophore hypothesis, the total cost should be close to the fixed cost, and far from the null cost. In addition, the higher null cost and the lower fixed cost represent the meaningful pharmacophore hypotheses generation. Along with three cost values, other statistical parameters such as a cost difference, RMSD, and r^2^_training_ were calculated. The cost difference is defined as a difference between the null cost and the total cost. A cost difference of 40~60 bits implies a 75~90% chance of representing a true correlation in the data [23,38]. The RMSD is a measure for the error between the experimental and estimated activity of the training set compounds. The significant pharmacophore model should have the higher cost difference, the higher r^2^_training_, and the lower RMSD.

#### 3.2.2. Test Set Method

The 32 compounds were used as the test set to validate the pharmacophore models. This method is used to investigate whether the generated pharmacophore model is sufficient to predict the biological activities of new compounds other than the training set compounds, and to classify them correctly in their activity scale. The biological activities of 32 test set compounds were predicted using the Ligand Pharmacophore Mapping with the Best/Flexible search option in DS. The r^2^_test_ was used as a primary criterion to determine the best pharmacophore model.

#### 3.2.3. Fischer Randomization Method

The Fischer randomization test was carried out to verify whether a high correlation exists between the chemical structures of the training set compounds and their biological activities. This method generates pharmacophore models by randomizing the biological activity data of the training set compounds, whereas using the same parameters used to develop the original pharmacophore model. During the pharmacophore hypotheses generation, if the hypothesis generated by a randomized dataset has similar or better various statistic values, including cost values, correlation, and RMSD, it means the original hypothesis has been generated by chance [22,23]. In this study, a 95% confidence level was selected for pharmacophore model validation, and 19 random hypotheses were constructed.

#### 3.2.4. Leave-One-Out Method

Finally, the pharmacophore model was validated by the LOO method. In this method, one compound in the training set was omitted from the building step of a new pharmacophore model, and its biological activity was predicted by that new model. This method can verify whether the generated pharmacophore model is strongly dependent on single particular training set compounds [22,23]. In this study, under the same parameter settings used for the generation of the original pharmacophore model, 20 pharmacophore models were generated by omitting one compound at a time from the 20 training set compounds.

### 3.3. Virtual Screening

The SDF files of 309,149 compounds were downloaded from the ZINC database, including both chemicals and natural products [32]. All the compounds in the ZINC database were primarily filtered based on the Lipinski’s rule of five [39], and the ADMET properties that were predicted by the ADMET Descriptors of DS. The selection criteria of Lipinski’s rule used in this study are as follows [39]: (i) less than five hydrogen bond donors; (ii) less than 10 hydrogen bond acceptors; (iii) a molecular weight of less than 500 Da; (iv) a logarithm of an octanol/water partition coefficient (Log P) value of less than 5. The selection criteria of ADMET used in this study are as follows [40,41,42,43,44,45]: (i) aqueous solubility level = 3 (yes, good) or 4 (yes, optimal); (ii) blood brain barrier (BBB) penetration level = 3 (low); (iii) intestinal absorption level = 0 (good); (iv) CYP2D6 = false (non-inhibitor); (v) hepatotoxicity = false (non-toxic). Conformers were generated for each molecule in the ZINC database using the Best conformer generation method that allows a maximum energy of 10 kcal/mol above that of the most stable conformation. Then, the best pharmacophore model validated using several different methods was used as a 3D query in database screening. The Ligand Pharmacophore Mapping with the Best/Flexible search option was applied to the database screening, in order to identify novel hit compounds that fit all the pharmacophore features. Finally, the retrieved compounds were further filtered by the criterion that compounds have estimated activity values of less than 100 nM.

### 3.4. Molecular Docking

The Libdock algorithm [46] in DS was used to perform molecular docking. Docking experiments on the final candidate sets of ligand-based virtual screening were carried out against the ANO1 protein. Cryo-EM structure complex of ANO1 with Ca^2+^ obtained at 3.75 Å was downloaded from the protein data bank (PDB ID: 5OYB) [28]. Protein preparation and minimization were carried out using the Prepare protein of DS. Hydrogen atoms were added to the protein–ligand complex under the CHARMM force field. All water molecules were removed, and the pH environment was adjusted to neutral. The active site was defined at a vicinity of the calcium-binding site with a 10 Å radius. The Libdock scores were obtained by the Libdock algorithm with the default setting except for calculating ligand conformations within an energy range of 10 kcal/mol above the global energy minimum. The docking pose and interaction between the ANO1 and the final hit compounds were analyzed using the Analyze Complexes panel of DS.

## 4. Conclusions

In this study, a ligand-based 3D-QSAR pharmacophore modeling with a subsequent molecular docking approach was employed to discover the novel lead candidates of ANO1 inhibitor. The best quantitative pharmacophore model, Hypo2, was generated based on the chemical features present in known ANO1 inhibitors with characterizing by the total cost (73.604), the cost difference (121.00), the RMSD (0.946), and the r^2^_training_ (0.969). Hypo2 has one HBA, one HBD, one HY, and one RA feature. The Hypo2 model was further assessed by the test set prediction, Fischer randomization, and leave-one-out method. The activity values of test set compounds were predicted well by Hypo2, resulting in a r^2^_test_ of 0.909. The other two methods also have revealed the solid validation results of Hypo2. Then, virtual screening of large chemical database (ZINC) was carried out using Hypo2. The retrieved hit compounds were filtered based on the estimated activity threshold of 100 nM, and these filtered 580 compounds were subjected to molecular docking for further characterization. Finally, two lead candidate compounds from the natural products space were identified with strong interactions with the calcium-binding site of ANO1. On the other hand, a docking study on the other putative active sites and a molecular dynamics simulation are needed to identify more reliable lead candidates. Moreover, further biological validation of the two lead candidates would be necessary to absolutely determine the inhibitory activity, as well as their binding mechanism on ANO1. However, to the best of our knowledge, this is the first study where both the ligand and structure-based approach were applied to identify a novel lead candidate of ANO1 inhibitors, although one pharmacophore model of CaCC_inh_-A01 and its derivatives was reported [47]. Therefore, the identified novel lead candidates and pharmacophore model in this study could be further employed in designing novel and potent ANO1 inhibitors in the future.

## Figures and Tables

**Table 1:** Statistical results of the top 10 pharmacophore hypotheses generated by HypoGen algorithm.

Hypothesis	Total Cost	Cost Difference ^a^	RMSD	Correlation (r^2^_training_)	Correlation (r^2^_test_)	Features ^b^
Hypo1	70.969	123.64	0.846	0.975	0.750	HBA, HBD, HY, RA
Hypo2	73.604	121.00	0.946	0.969	0.909	HBA, HBD, HY, RA
Hypo3	80.233	114.37	1.259	0.945	0608	HBA, HBA, HY, RA
Hypo4	81.841	112.77	1.324	0.939	0.441	HBA, HBD, HY, RA
Hypo5	83.504	111.10	1.400	0.931	0.362	HBA, HBD, HY, RA
Hypo6	85.290	109.32	1.466	0.924	0.449	HBA, HBD, HY, RA
Hypo7	85.999	108.61	1.444	0.927	0.413	HBA, HBD, HY, RA
Hypo8	86.400	108.21	1.503	0.920	0.702	HBD, HY, RA
Hypo9	86.982	107.63	1.482	0.923	0.634	HBA, HBD, HY, RA
Hypo10	87.091	107.52	1.436	0.927	0.584	HBA, HBD, HY, RA

Null cost = 194.608; Fixed cost = 63.804; Configuration cost = 15.343; ^a^ Cost difference = null cost − total cost; ^b^ Abbreviations for features: HBD, hydrogen-bond donor; HBA, hydrogen-bond acceptor; HY, hydrophobic; RA, ring aromatic.

**Table 2:** Experimental and estimated IC_50_ values of the training set compounds based on the best pharmacophore hypothesis, Hypo2.

Compound	IC_50_ (μM)		Error ^b^	Activity Scale ^c^	
	Experimental ^a^	Estimated		Experimental	Estimated
1	0.107	0.128	+1.2	++++	++++
2	0.194	0.119	−1.6	++++	++++
3	0.481	0.511	+1.1	++++	++++
4	1.8	2.8	+1.6	+++	+++
5	2.8	5.9	+2.1	+++	+++
6	2.8	2.8	−1	+++	+++
7	3.2	5.9	+1.8	+++	+++
8	3.5	3.9	+1.1	+++	+++
9	4.4	6.7	+1.5	+++	+++
10	5.7	5.5	−1	+++	+++
11	6.2	5	−1.2	+++	+++
12	7.9	6.1	−1.3	+++	+++
13	9	9.6	+1.1	++	++
14	13.3	9.2	−1.4	++	++
15	16.3	9.4	−1.7	++	++
16	18	21.3	+1.2	+	+
17	23	9.2	−2.5	+	++
18	25.9	26.7	+1	+	+
19	27	31.8	+1.2	+	+
20	29.2	26.6	−1.1	+	+

^a^ References [10,13,30,31]; ^b^ Positive value indicates that the estimated IC_50_ is higher than the experimental IC_50_; negative value indicates that the estimated IC_50_ is lower than the experimental IC_50_; ^c^ Activity scale: IC_50_ < 1 μM (Most active, ++++); 1 ≤ IC_50_ < 8 μM (Active, +++); 8 ≤ IC_50_ < 18 μM (Moderately active, ++); IC_50_ ≥ 18 μM (Inactive, +).

**Table 3:** Experimental and estimated IC_50_ values of the test set compounds based on the best pharmacophore hypothesis, Hypo2.

Compound	IC_50_ (μM)		Error ^b^	Activity Scale ^c^	
	Experimental ^a^	Estimated		Experimental	Estimated
1	0.301	0.111	−2.7	++++	++++
2	0.512	0.195	−2.6	++++	++++
3	1.02	0.399	−2.6	+++	++++
4	2.9	3.3	+1.1	+++	+++
5	3.9	3.6	−1.1	+++	+++
6	4.4	5.7	+1.3	+++	+++
7	4.9	6.7	+1.4	+++	+++
8	5.2	3.2	−1.6	+++	+++
9	5.2	6.4	+1.2	+++	+++
10	5.4	4.4	−1.2	+++	+++
11	5.6	6.7	+1.2	+++	+++
12	5.7	4.4	−1.3	+++	+++
13	6	4.9	−1.2	+++	+++
14	6.3	5.6	−1.1	+++	+++
15	6.3	9.2	+1.5	+++	++
16	6.5	3.3	−2	+++	+++
17	7.7	6.6	−1.2	+++	+++
18	7.9	7.9	−1	+++	+++
19	8.4	10.6	+1.3	++	++
20	10.8	9.5	−1.1	++	++
21	11.2	13.3	+1.2	++	++
22	12	6.1	−2	++	+++
23	12.3	6.6	−1.9	++	+++
24	13.1	9.1	−1.4	++	++
25	15	11	−1.4	++	++
26	15.6	9.3	−1.7	++	++
27	16	14.5	−1.1	++	++
28	17.3	9.7	−1.8	++	++
29	21.2	28.4	+1.3	+	+
30	22	11.4	−1.9	+	++
31	23.5	31.4	+1.3	+	+
32	28.7	31.3	+1.1	+	+

^a^ References [10,13,30,31]. ^b^ Positive value indicates that the estimated IC_50_ is higher than the experimental IC_50_; negative value indicates that the estimated IC_50_ is lower than the experimental IC_50_. ^c^ Activity scale: IC_50_ < 1 μM (Most active, ++++); 1 ≤ IC_50_ < 8 μM (Active, +++); 8 ≤ IC_50_ < 18 μM (Moderately active, ++); IC_50_ ≥ 18 μM (Inactive, +).

**Table 4:** 2D chemical structures of the final hit compounds with corresponding estimated scores.

Compound	Structure	Fit Value	Estimate (μM)	Libdock Score
ZINC8643627	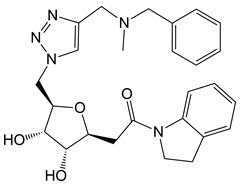	6.29	0.052	162.29
ZINC225516955	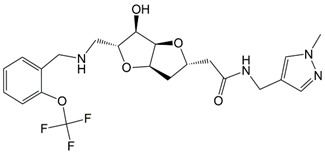	6.04	0.092	160.92

## Abbreviations

ZINC	ZINC Is Not Commercial database
ADMET	Absorption, Distribution, Metabolism, Excretion, and Toxicity
MONNA	*N*-((4-methoxy)-2-naphthyl)-5-nitroanthranilic acid
FDA	The Food and Drug Administration
ANO2	Anoctamin 2
CFTR	Cystic fibrosis transmembrane conductance regulator
ENaC	Epithelial sodium channel
PDE4	Type 4 cyclic adenosine monophosphate (cAMP) phosphodiesterase
BACE1	β-Site amyloid precursor protein-cleaving enzyme
AKR1B10	Aldo-keto reductase family 1 B 10
CYP2D6	Cytochrome P450 2D6
